# Sub-surface magma movement inferred from low-frequency seismic events in the off-Nicobar region, Andaman Sea

**DOI:** 10.1038/s41598-020-78216-2

**Published:** 2020-12-04

**Authors:** K. K. Aswini, Pawan Dewangan, K. A. Kamesh Raju, V. Yatheesh, Pabitra Singha, Lalit Arya, T. Ramakrushana Reddy

**Affiliations:** 1 CSIR-National Institute of Oceanography, Dona Paula, Goa 403004 India; 2 School of Earth, Ocean and Atmospheric Sciences, Goa University, Taleigao Plateau, Goa 403206 India; 3 National Centre for Polar and Ocean Research, Ministry of Earth Sciences, Vasco-da-Gama, Goa 403804 India

**Keywords:** Natural hazards, Ocean sciences, Solid Earth sciences

## Abstract

Monitoring volcanic activity along the submarine volcanoes that are usually induced by subsurface magmatism is a challenge. We present fresh set of Ocean Bottom Seismometer (OBS) data that shows geophysical evidence indicative of subsurface magmatism along the submarine volcanoes in the off Nicobar region, Andaman Sea. In this region, we observed for the first time, hybrid very long-period earthquakes documented by passive OBS experiment. These events were initiated by high-frequency (5–10 Hz) with a clear onset of P-phase followed by low-frequency (0.01–0.5 Hz) oscillations in the range of 300–600 s with a prominent high-frequency (10–40 Hz) hydro-acoustic phase. A total of 141 high-frequency events were detected on 21st and 22nd March 2014 out of which 71 were of low-frequency oscillations. These events are distributed in the northwest–southeast direction along the submarine volcanic arc and Seulimeum strand of Great Sumatra fault. Off Nicobar region has been witnessing frequent earthquake swarms since 26th December 2004 tsunamigenic Sumatra earthquake. These swarms occurred in January 2005, March and October 2014, November 2015 and March 2019. The occurrence of low-frequency earthquakes and prominent hydro-acoustic phase are suggestive of sub-surface tectonic and magmatic influence. We propose that upward movement of magma pulses from deeper magma reservoir to the shallow magma chamber activated the strike-slip movement of sliver faults and induced earthquake swarms in the off Nicobar region.

## Introduction

Earthquakes associated with volcanic activity provide insights about the dynamics of active magmatic systems. Such earthquakes can be classified based on the physical processes and characteristics of the waveforms: volcano-tectonic (VT) events, volcanic tremors and low frequency events (long-period events (LPEs), very long-period events (VLPEs), and hybrid events.)^1–3^. Volcano-tectonic events arise out of shear failure and are induced by stress changes caused by magma movement^4^. LPEs are characterized by emergent waveforms with no distinct P- and S- phases and show dominant lower frequency range from 0.5 to 5 Hz^5^. VLPEs are long duration signals (3 to 100 s or longer) with a dominant frequency range of 0.01 to 0.5 Hz^2^. Hybrid earthquakes are mixed earthquakes characterized by high frequency onset followed by a long period non-dispersive harmonic coda. The high-frequency event is considered to be of tectonic origin that may have actuated the long-period event^4^. Volcanic tremors are continuous signals similar to LPEs lasting from minutes to days^1,2^. Due to shallow nature, volcano-tectonic events that occur underwater often generate hydro-acoustic waves (observed as T-waves in continental stations) that propagate through the SOFAR (Sound Fixing and Ranging) channel in deep-ocean, and can be detected by broadband hydrophones. Such phases are commonly used to monitor submarine volcanic activity^6–10^.

LPEs, VT and earthquake swarms are well documented in literature and seem to be associated with active volcanoes^4,11–14^. Earthquake swarms are observed in submarine volcanoes such as Lohi Seamount^15^, Okinawa Trough in East China Sea^16^, Axial volcano Juan de Fuca Ridge^17,18^, and Oceanic spreading centers^19,20^, these swarms are related to subsurface magma movement. The VT events and LPEs observed near Mayotte in the western Indian Ocean are inferred to be associated with sub-surface magma movement^21^.

Earthquake swarms are observed frequently in the off Nicobar region following the Tsunamigenic 26th December 2004 megathrust earthquake. The swarm in January 2005 is the most energetic swarm ever recorded globally^22–24^. Stress changes due to the megathrust event, strike-slip faulting and submarine volcanism in the off Nicobar region are some of the possible reasons suggested for the occurrence of 2005 swarm^23–27^. Bathymetry data show the presence of cratered submarine volcanoes^23^ in the off Nicobar region; however, no other geophysical evidence of recent magma movement has been reported from this region.

**Figure 1:**
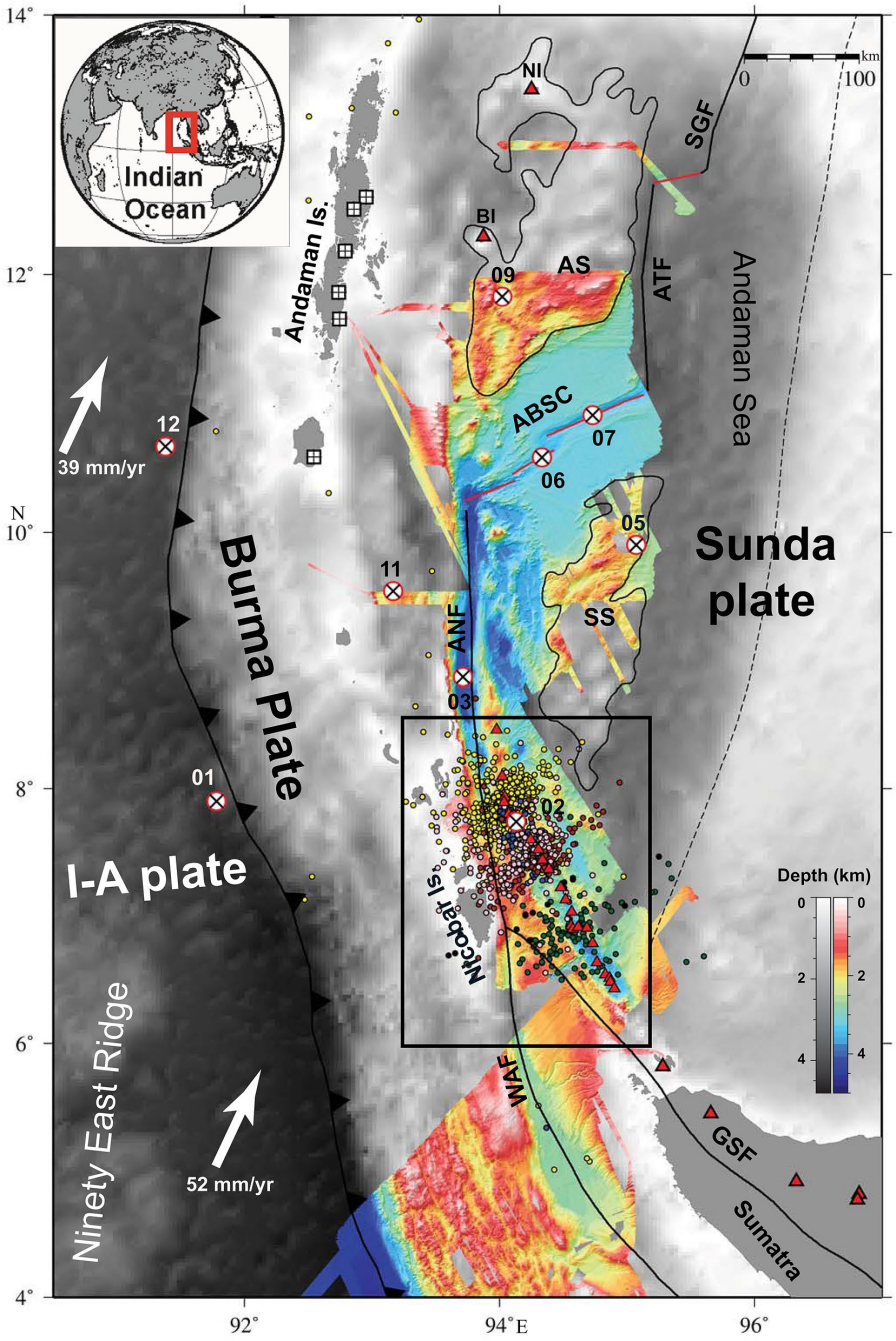
Tectonic framework of the Andaman Sea presented on a gray shaded bathymetric image^62^ with available high-resolution bathymetry data acquired by CSIR-NIO along with published data of NW Sumatra region between 5°N and 6.5°N^63^. Black squares with cross denote broad-band seismic stations from the ISLANDS network^39^. Red circle with a black cross represents the Ocean Bottom Seismometer deployments^31^. Indo Australian plate (I-A plate), Great Sumatra Fault (GSF), West Andaman Fault (WAF), Andaman–Nicobar Fault (ANF), Andaman Backarc Spreading Centre (ABSC)^28^, Andaman Transform Fault (ATF), Sagaing Fault (SGF), Sewell Seamount (SS) and Alcock Seamount (AS) are marked. The dashed black line represents the ocean-continent boundary proposed by Curray^29^. Red triangles represent volcanoes. Black box represents the study area off Nicobar Island.

The Andaman–Nicobar–Sumatra subduction zone is characterized by a chain of volcanoes starting with Sumatra volcanoes in the south, to submarine volcanoes in the middle, to sub-aerially exposed Barren and Narcondum volcanoes in the north, and together they define the inner volcanic arc^23^. The trench-parallel motion occurring between the Indo-Australian plate and the Southeast Asian plate is primarily accommodated by the Sliver fault system, which includes the Sagaing Fault (SGF) in the north, Andaman Transform Fault (ATF) and Andaman Back-arc Spreading Centre (ABSC) in the middle, and Andaman Nicobar Fault (ANF), West Andaman Fault (WAF), the Great Sumatra Fault (GSF) in the south^28–31^ (Fig. 1). In the offshore region, the GSF splits into two strands: the Aceh Fault (AF) and the Seulimeum Fault (SF)^32,33^. The 2004 and 2005 megathrust events imparted static Coulomb stress within the major fault systems of the Andaman–Nicobar–Sumatra subduction zone and modulated the seismicity^25,34,35^. The stress inhibited failure along the SGF and promoted failure along the ABSC, ANF and the GSF systems. After megathrust events, the imparted Coulomb stress increased by almost 20 bar in the northern segment of the GSF, which makes the region susceptible to future earthquakes^31,35^. After the January 2005 off Nicobar earthquake swarm^23,24^, this region has experienced four distinct bouts of earthquake swarms in March 2014, October 2014, November 2015 and March 2019 (Fig. 1).

CSIR-National Institute of Oceanography (CSIR-NIO), Goa, India conducted a passive Ocean Bottom Seismometer (OBS) experiment from 22nd Dec 2013 to 15th May 2014 by deploying 12 OBS (OBS01-OBS12) receivers at selected locations in the Andaman Sea (Fig. 1). The analysis of ambient and instrument-related noise is reported by Dewangan et al. (2018)^36^ and Reddy et al. (2020)^37^ whereas local seismicity is reported by Singha et al. (2019)^38^. To understand the nature of swarms in the off Nicobar region, we deployed one of the OBSs (OBS02) in this region (Fig. 1). In the present study, we report for the first time the occurrence of low-frequency (very long-period) earthquakes on 21st and 22nd March 2014, a suggestive of subsurface magma movement in the off-Nicobar region.

## Data and methodology

Twelve broadband, three-component, ocean bottom seismometers (OBS) were chartered from K.U.M. Umwelt-und Meerestechnik, Keil GmbH and deployed at selected locations in the Andaman Sea from 22nd Dec 2013 to 15th May 2014 (Fig. 1). The details of the passive OBS experiment in the Andaman region are discussed in Dewangan et al. (2018)^36^ and Singha et al. (2019)^38^. In the present study, we considered the OBS data of 21st and 22nd March 2014, when an earthquake swarm occurred in the off Nicobar region. We have also integrated the data from the Andaman ISLANDS network (CBY, BAKU, BARA, SBY, PBA, HUTB, Fig. 1)^39^, and the land station data (from Incorporated Research Institution for Seismology (IRIS) Data Management Centre).

**Figure 2:**
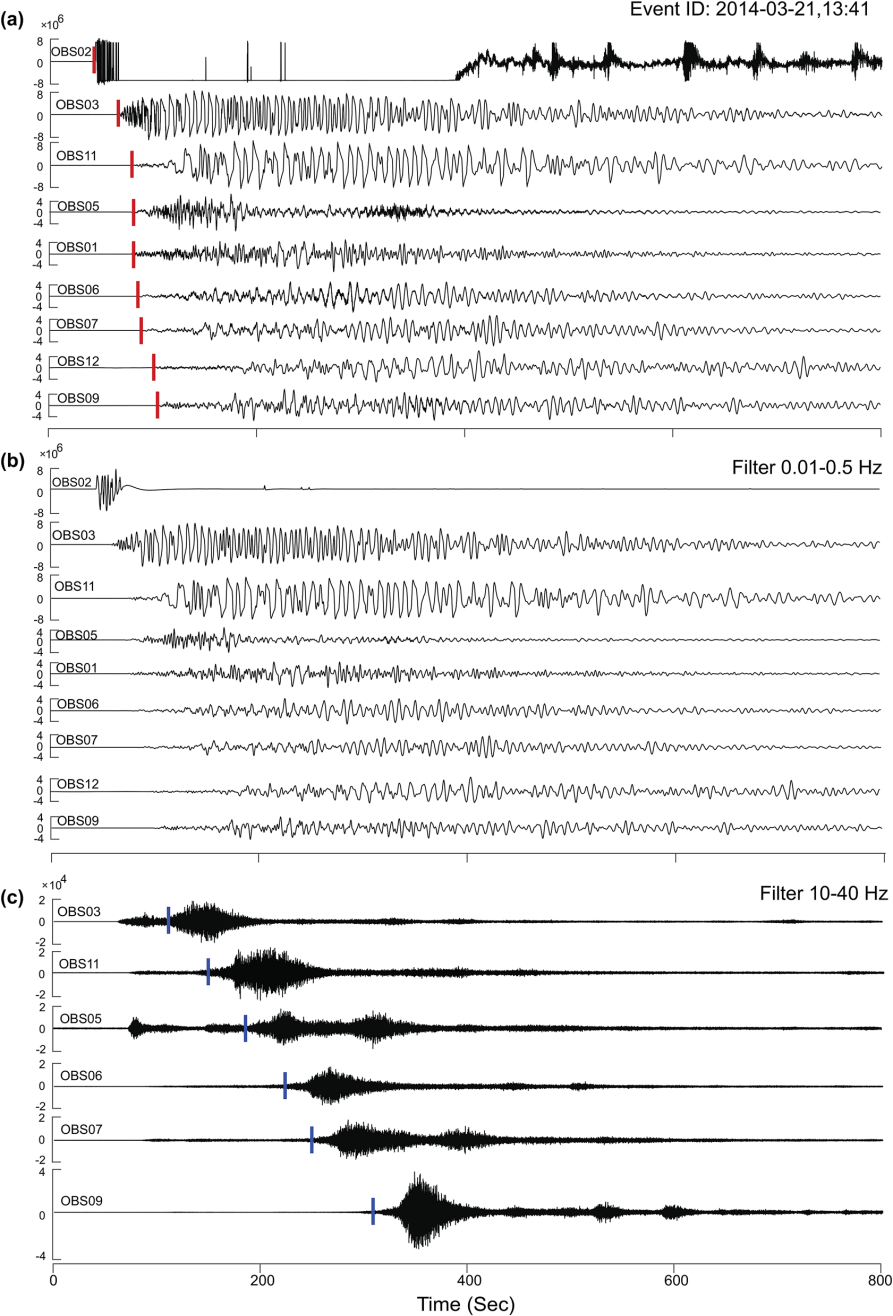
Normalised wave forms of 21st March 2014 event. (**a**) Hybrid long-period event recorded at all OBS stations. The red bars indicate the p-phase (IP). (**b**) Low-frequency component of the 21st March long period event in the frequency band 0.01–0.5 Hz. (**c**) High-frequency (10–40 Hz) hydro-acoustic wave associated with long period event. The blue bars indicate the hydro-acoustic arrival phase. Location of the OBS stations are given in Fig. 1.

**Figure 3:**
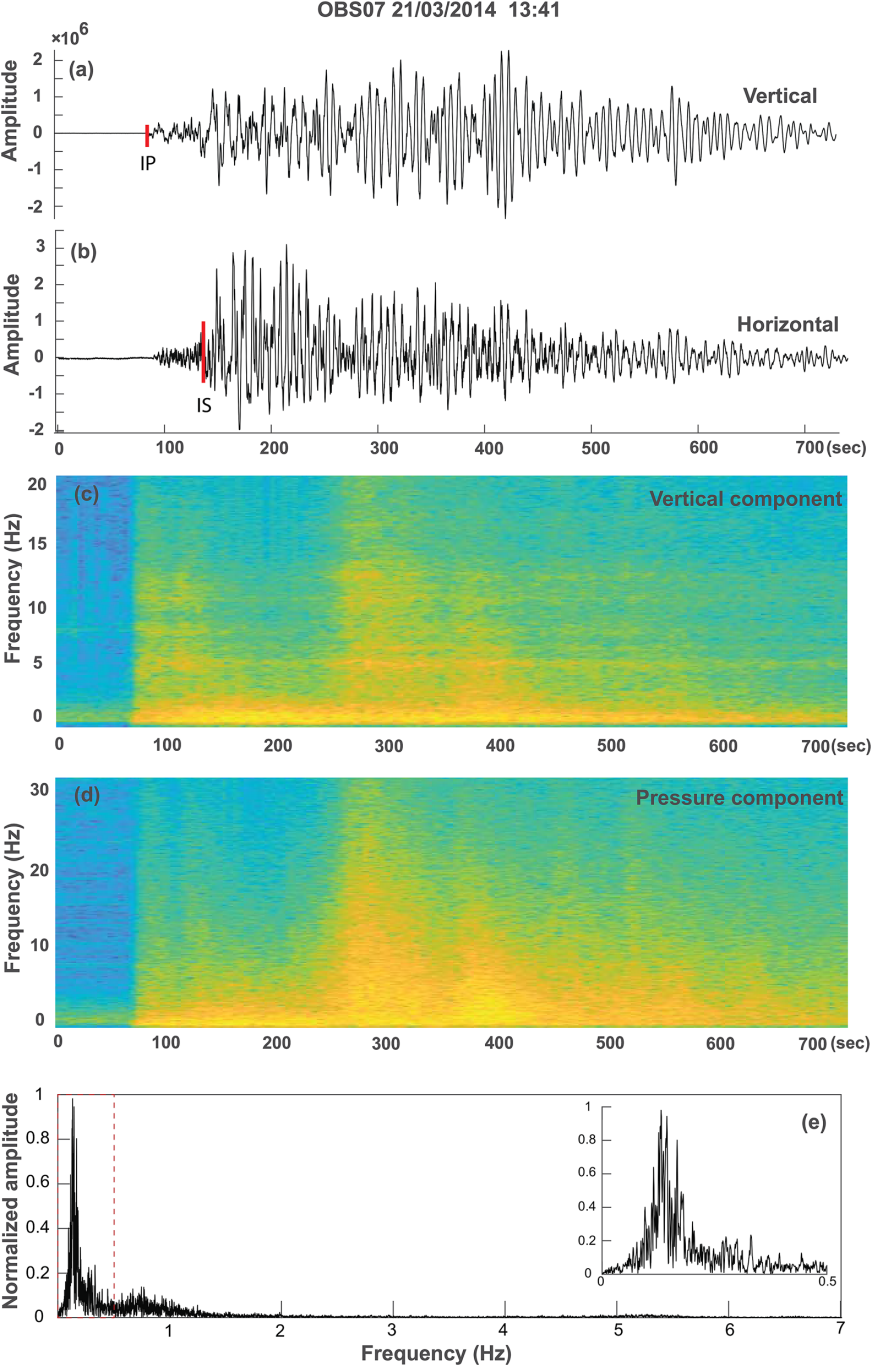
Examples from 21st March main event from OBS07 station. (**a**) Vertical component of the event taken for spectral analysis. Red bar indicates p-phase (IP). (**b**) Horizontal component of the event. red bar indicates s-phase (IS). (**c**) Spectrogram of vertical component. (**d**) Spectrogram of pressure component highlighting high frequency hydroacoustic waves. (**e**) Velocity spectra of the event. Inset shows the enlarged view of 0–0.5 Hz part of the spectra.

We used SEISAN software to identify the earthquake events in different frequency bands. We extracted and registered the events occurred on 21st and 22nd March after the main event from OBS and all available ISLAND network and land stations. The first motions of these events show mixed compression and dilatation polarities at different seismic stations. The high-frequency impulsive and clear P-phase onset helped us to pick the P-wave arrival time and first motion polarity. The initial waveforms have a clear high-frequency P-phase onset. The seismograms are characterized by a high–frequency onset earthquake, which is followed by low-frequency long-duration oscillations, and a representative event (6.5 M_w_) on 21st March at 13:41 UTC is shown in Fig. 2a. The high-frequency onset shows a frequency band of 1–10 Hz with a clear P-phase onset followed by low-frequency (0.01–0.5 Hz) oscillations for 600 s (Fig. 2b). The duration of the event is significantly large as compared to that of local tectonic earthquakes. We also observed hydro-acoustic phase (T-waves, 10–40 Hz frequency) associated with the events on 21st and 22nd March in the pressure component of OBS data (Fig. 2c). Time–frequency analysis of waveform data depicts a hybrid earthquake event, a representative event from OBS07 is shown in Fig. 3. We have also shown waveform and frequency spectra of a smaller magnitude earthquake (M_L_ 4.1) as one more example (see Supplementary Figs. S1a, S2).

**Figure 4:**
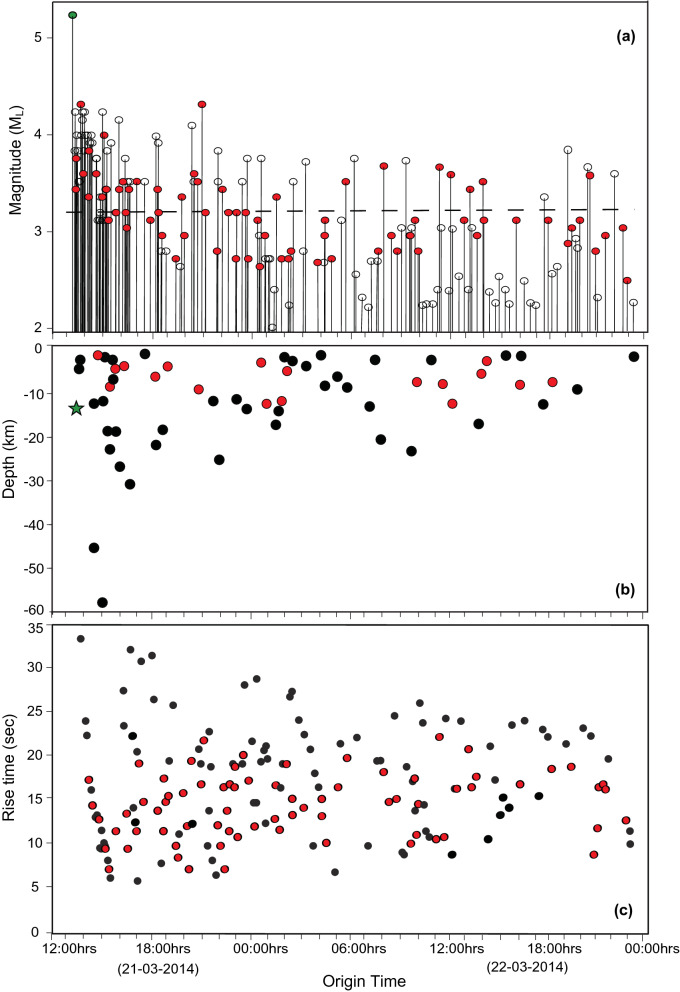
Magnitude, depth and rise time of March 2014 swarm. (**a**) Earthquake magnitude plotted against origin time of the earthquake swarm events. Black dotted line is the average magnitude value excluding the main event. (**b**) Hypocenter distribution for the events detected on 4 or more station using hypocenter v. 3.2 program. Depths that are not constrained due to inadequate azimuthal coverage are not plotted in the figure, Green star denotes the M_W_ 6.5 event and filled red circles are the events with depth less than 5 km, other events are in black. (**c**) T-phase rise time plotted against the origin time of the events. Red color filled circles indicate VLPEs.

The picked arrival times were used for locating the earthquakes using Hypocent v.3.2 program^40^ and the minimum 1D velocity model established by Singha et al. (2019)^38^. We estimated the location error ellipse using a covariance matrix, as defined in the Hypocent program. Local magnitude is calculated using the amplitude picked on an equivalent Wood-Anderson seismograph (Fig. 4a) and located the hypocenter for the events detected on 4 or more station using hypocenter v. 3.2 program^40^ (Fig. 4b). We also located these events using hydroacoustic phase with a water velocity of 1.5 km/s. We have computed rise time of hydro-acoustic wave (Fig. 4c) using the technique developed by Schreiner et al. (1995)^41^. We have determined focal mechanism of 4 representative high magnitude (> M_L_ 4.0) earthquakes by picking the first motion P-wave polarity on more than 30 stations using HASH program^42^.

## Results

A high magnitude event (M_w_ 6.5) occurred on 21st March 2014 at 13:41 UTC (Fig. 2) followed by an earthquake swarm that lasted till 22nd March 2014 are documented in the passive OBS data. The epicenter of the earthquakes lie close to OBS02, and the events were recorded by all OBS and ISLAND stations. The initial onset of this event is dominated by a high-frequency component followed by long-duration low-frequency oscillations (Fig. 3a–d). A very-high frequency signal is observed in the middle of low-frequency oscillations in the pressure component (Fig. 3d). The spectrum of the vertical component shows a spectral peak at 0.13 Hz (Fig. 3e). We performed a similar analysis for all OBS, ISLAND and continental stations, and observed comparable waveform and spectral characteristics. A detailed description of various components of these events is as follows.

### High-frequency onset earthquakes from the off Nicobar region

**Figure 5:**
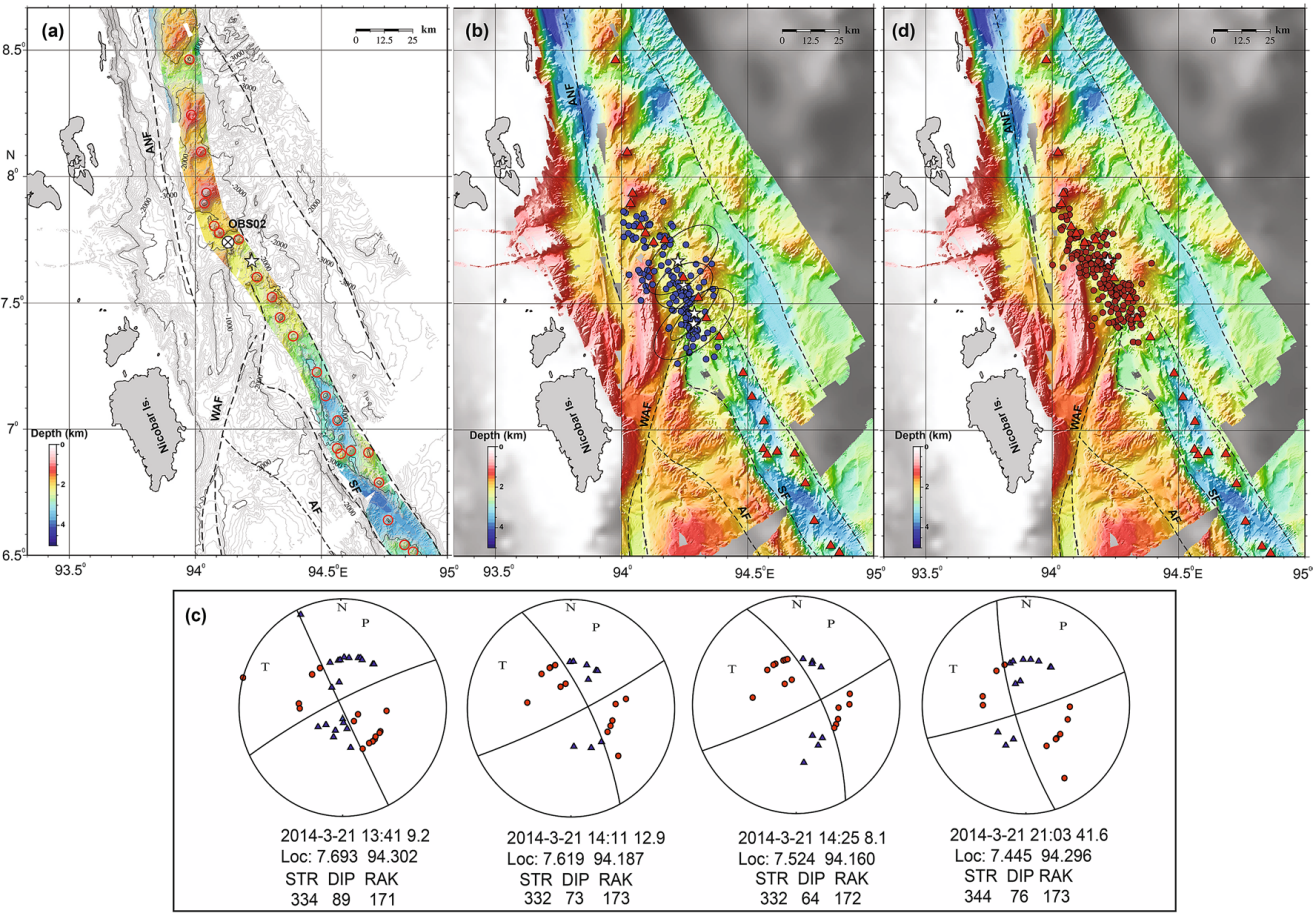
High resolution bathymetry, earthquake swarm locations and focal mechanism solutions. (**a**) Colour coded bathymetric image highlights the identified volcanoes (red circles) with 100 m interval. White star indicates 21 March 2014 main event. (**b**) 21st March 2014 main event and the epicenters of the high-frequency onset events plotted on the multibeam bathymetry map. White stars indicate high magnitude events selected for fault plane solution and error analysis. Black ellipses represent the error ellipse. Red triangles represent identified volcanoes. (**c**) Fault plane solutions of the 21st March 2014 main event and some of the larger events present in the swarm. The filled red circles denote the positive motion or compression while the filled blue triangles denote the negative motion or the dilatation. The solution gives strike, dip and rake values of the fault. (**d**) Location of events determined from the hydro-acoustic phase in the hydrophone component of the OBS.

A total of 141 local events are detected on three or more stations on 21st and 22nd March 2014. The onset is marked by a high–frequency (1–10 Hz) earthquake characterized by an impulsive waveform with a clear P-phase (Fig. 3a), and an S-phase (Fig. 3b). The local magnitudes (M_L_) vary from 2.0 to 5.2. We have plotted the magnitude vs time of these two days events (Fig. 4a). This analysis suggests that after the main event (Mw 6.5), the sequence does not follow the classical main shock-aftershock pattern. We observe an initial decay of magnitude with time, but high magnitude events are present in the middle and towards the end of the sequence, suggesting that the earthquake cluster can be classified as swarm. The focal depths of earthquakes estimated using both OBS and ISLAND network for most of the events, fall in the range of 30 to 1 km (Fig. 4b). However, the accuracy of depth estimates is limited due to the number of receivers in the source area and inadequate azimuthal coverage (< 180°). Epicenters of these events are located near OBS02, and are distributed linearly in the northwest-southeast direction (Fig. 5b). The major axes of error ellipses are almost perpendicular to the linear trend and oriented in the southwest-northeast direction. High-resolution multibeam bathymetry data show the presence of several submarine volcanoes demarcating the inner volcanic arc (Fig. 5a). The map also highlights major fault systems like ANF, WAF, and SF that comprise the sliver fault. It is interesting to note that the epicentres follow the trend of SF (Fig. 5b).

The focal mechanism of the 21st March main event suggests a vertical right-lateral strike-slip fault with strike, dip and rake values of 334°, 89° and 171°, respectively. The fault parameters correlate with local geology (strike of SF, ~ 330°, Fig. 5a). The orientation of P-axis (pressure or compressional axis) is SSW and T-axis (tension axis) is ESE. The focal mechanisms of the other three significant events are similar to the main event and show a vertical right-lateral strike-slip fault (Fig. 5c).

### Long-duration low-frequency events

High-frequency earthquake events followed by long-duration (300–600 s) oscillations (VLPEs) are documented in all the deployed OBS data. The VLPEs are identified in 71 events out of the total 141 events observed during 21–22 March 2014. The waveform has a dominant frequency band of 0.01–0.5 Hz (Fig. 2b). The duration of these events is unusually large as compared to that of tectonic earthquakes (see Supplementary Fig. S1). Magnitude of VLP events varied from 2.6 to 4.2 M_L_ (Fig. 4a) and the depth varied from 1 to 12 km (Fig. 4b). The VLPEs observed from the off Nicobar region can be classified as hybrid events with clear high-frequency onset earthquakes and mixed first motion polarities. Such VLPEs are often associated with magmatism, which might have been initiated by the 6.5 M_W_ main shock.

### Hydro-acoustic phase arrivals

Hydroacoustic phase arrivals (10–40 Hz) are detected primarily by a hydrophone attached to an OBS unit (Fig. 2c). These events are observed only on OBS stations located in the Andaman Sea (OBS02, OBS03, OBS05, OBS06, OBS07, OBS09, OBS11). They are not detected by island and continental stations as well as the OBS stations located on the subducting Indian plate (OBS01 and OBS12, Fig. 1), because the Nicobar-Andaman arc lies between those stations and the swarm earthquakes. These events travel through the water column with a velocity of 1.5 km/s and are referred to as the hydro-acoustic phase. In general, acoustic waves generated by a submarine volcano or shallow earthquakes can travel to great distance (~ 15,000–16,000 km) in the open ocean through a low-velocity layer known as SOFAR channel^10,43,44^. In the present study, the OBSs located in deep waters (> 1500 m) detected the hydro-acoustic phase. The phase is preserved due to the conservation of energy owing to multiple total internal reflections^45^. The hydro-acoustic waveforms are emergent with no clear onset of P-phase and show long durations (> 100 s) (Fig. 3d).

A total of 181 hydro-acoustic phase detected on four or more OBS stations are documented, these are similar to the T-phase detected on land. The spatial distribution of epicentres shows a linear trend in the northwest-southeast direction along the submarine volcanoes (Fig. 5d). The hydro-acoustic phase is closely associated with high-frequency earthquakes and all the 141 earthquakes generated the hydro-acoustic phase; however, the pressure component detects more hydro-acoustic phase than the high-frequency onset events. Low magnitude seismic events are too weak to be detected by at least three stations, however the hydroacoustic phase associated with these events travel long distances as the water column is less attenuating than the oceanic crust and are detected by pressure sensor, resulting in detection of additional 40 hydroacoustic events.

## Discussion

The off Nicobar region has been witnessing frequent earthquake events after the 26th December 2004 tsunamigenic earthquake. A highly energetic earthquake swarm (259 events with m_b_ > 5) occurred after the tsunamigenic earthquake from 26th to 31st January 2005 in the off Nicobar region^22,23^. This swarm occurred between the ANF and the SF showed both strike-slip and normal faulting suggesting a volcano-tectonic origin^23,24^. There was a period of quiescence of earthquake swarms near Nicobar Island from 2005 to 2014 until a major hybrid LPE occurred on 21st March 2014, followed by earthquake swarms in October 2014, November 2015 and March 2019.

In the present study, we report for the first time the presence of hybrid VLPEs from off Nicobar region (dominant frequencies lower than 0.2 Hz). Hybrid VLPEs are often associated with subsurface magma movement and have been documented from active volcanoes such as Redoubt Volcano^1,2,4^ and Soufriere Hills Volcano on Montserrat^11^. The hybrid earthquake swarms at Redoubt Volcano are also characterized by long-period spectra with more pronounced high frequency onset comparable to VT events^1,4,13^. These events display mixed first motion polarities and are generated by brittle faulting of weak zone intersecting fluid-filled crack, and thus involve both double-couple and volumetric components^1,13^. The events observed in the off Nicobar region also show mixed characteristic of both LP and VT events. Dike induced hybrid earthquake swarms of Afar region are suggested to be good indicators of shallow fault rupture and the associated low frequency spectral content is indicative of active fluids in shallow subsurface^46^. VLP events (dominant frequencies lower than 0.2 Hz) similar to the Nicobar swarm are related to the displacement of material such as magma or gas^47,48^. Brittle rock fracture and a combined tensile and shear response of the crust in response to pulses of shallow magmatic intrusion were suggested by Pallister et al. (2010)^49^ based on the analysis of volcanic VLPEs associated with some of the high-frequency earthquakes. Although the reported^49^ duration of waveform (~ 100 s) was much smaller than that reported from Nicobar region (~ 300 s) we believe that the underlying mechanism is same. Recently, Cesca et al. (2020)^21^ observed 7000 VT events and 400 VLPEs near Mayotte region, the western Indian Ocean from the global network stations. VLPEs observed in the Mayotte region were associated with deep magma reservoir. VT events and some VLPEs occurred in a specific location near to the reservoir were suggested to be the indicators of vertical dike propagation.

**Figure 6:**
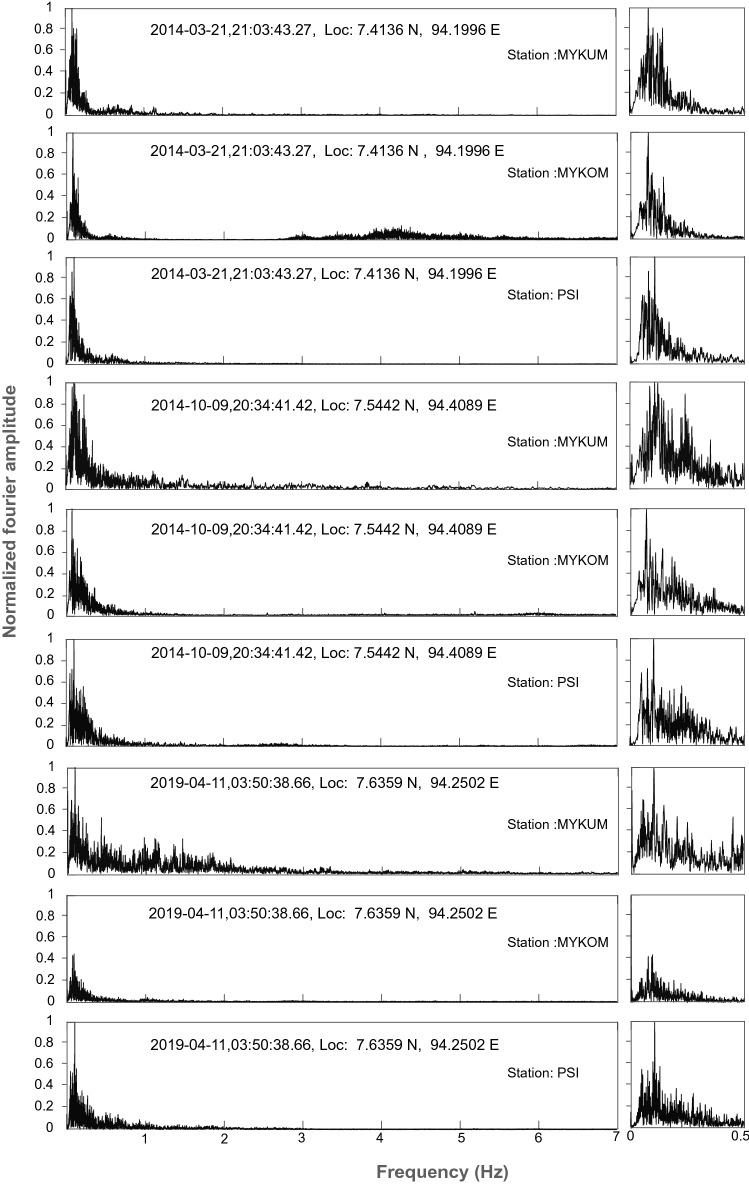
Example of velocity spectra for single event with similar magnitudes, performed for March 2014, October 2014 and April 2019 swarm data from nearby continental stations (PSI, MYKUM and MYKOM). Enlarged view of 0 to 0.5 Hz part of the velocity spectra is shown on the right side.

**Figure 7:**
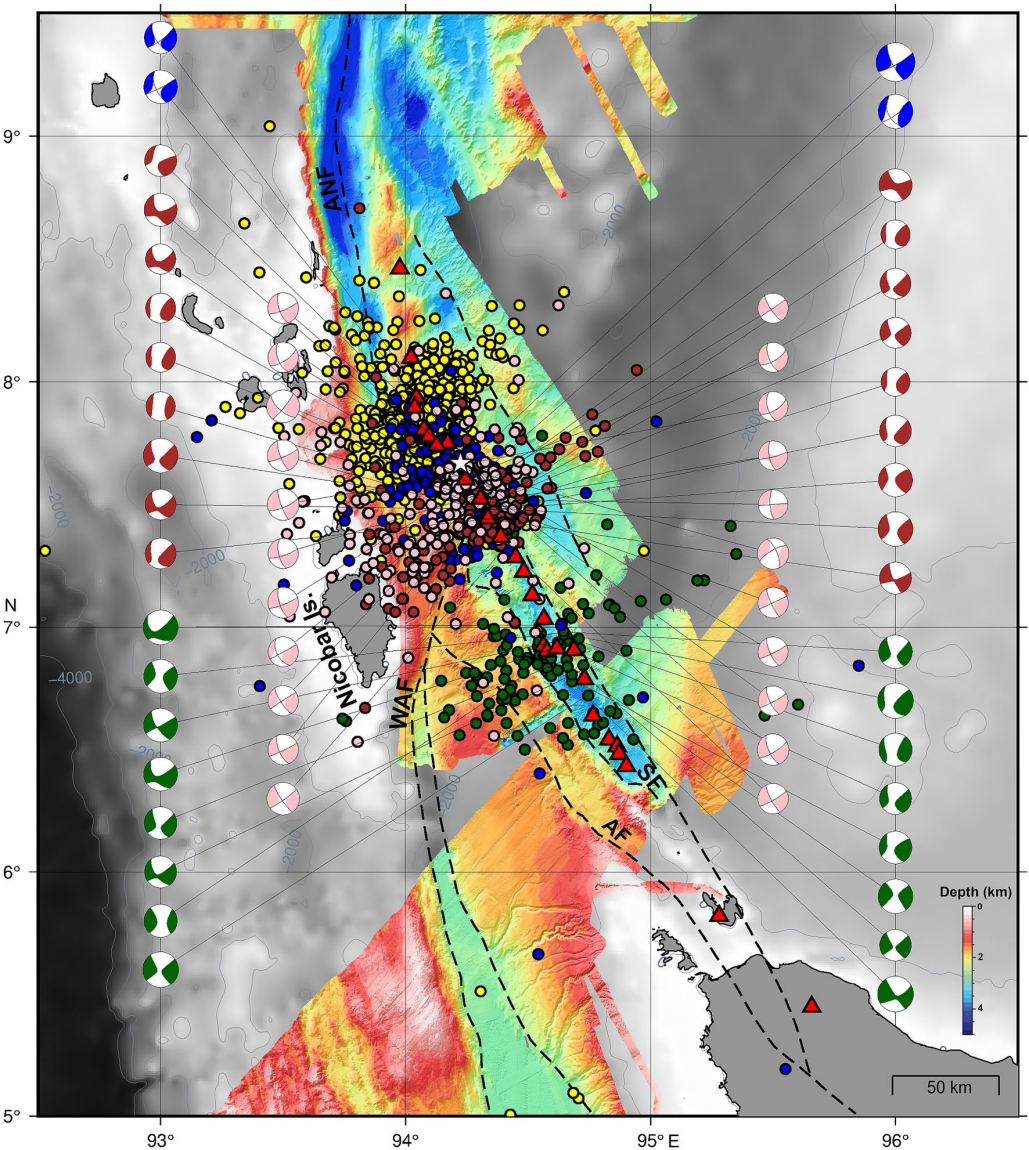
Earthquake swarms occurred off Nicobar region in Andaman Sea. Yellow circles indicate 2005 swarm, white star indicates the 21 March 2014 main shock, blue circles indicate 2014 aftershocks, brown circles indicate October 2014 swarm, green circle indicates November 2015 swarm and pink circle indicates March 2019 swarm. Figure shows corresponding fault plane solutions of the earthquakes.

In our study, the epicentres of hybrid VLPEs are distributed in the northwest-southeast direction along the inner volcanic arc in the vicinity of SF. The off Nicobar region has witnessed earthquake swarms in October 2014, November 2015 and March 2019. The spectral analysis of waveform data of other earthquake swarms from nearby continental stations (PSI, MYKUM and MYKOM), provided by IRIS Data Management Centre, show characteristics similar to that of March 2014 VLPEs (Fig. 6) with a well-defined peak at around 0.1 Hz. We are unable to confirm VLPEs on distant continental stations as inelastic attenuation may also yield low-frequency signal which may resemble VLPEs. Recurrence of these VLPEs suggests subsurface or may be surface magmatic activity along the inner volcanic arc. It is interesting to note that the focal mechanisms of 2014, 2015, 2019 swarms, obtained from GCMT (Global Centroid Moment Tensor, https://www.globalcmt.org/) catalogue, also suggest strike-slip fault earthquakes (Fig. 7). These solutions are the representation of the earthquakes with magnitude greater than 4. We do not have focal mechanism solution for low magnitude events in the swarm. A denser local network is required for measuring small magnitudes earthquakes, which may have volumetric component that is expected during dike intrusion. The strike-slip faulting and VLPEs are closely related to earthquake swarms observed in the off Nicobar region.

In the Andaman–Nicobar subduction zone, a shear component of ongoing tectonic movement is mostly accommodated by overriding Burmese plate as a consequence of oblique convergence^31,50–52^. Previous studies in the convergent boundaries suggest that the occurrence of magmatic arcs are characterized by strike-slip motion due to strain partitioning accommodated by oblique subduction of subducting plate^53,54^. Towards the northern part of Sumatra Island, a chain of volcanoes aligns with the Seulimeum strand of the GSF as observed in the high-resolution bathymetry data (Fig. 5a). The presence of the sliver strike-slip fault system in the off Nicobar region might have facilitated magma movement through vertical dykes aligned parallel to the principal stress axis. Hence, strike-slip faulting and subsurface magma movement are closely associated; however, it is difficult to establish whether the strike-slip faulting stimulated the subsurface movement of magma or vice versa. Kundu et al. (2012)^24^ computed the b-value (1.09 and 1.65) and reported bimodal distribution of frequency-magnitude relation for the 2005 swarm, indicating that the swarm is governed by both tectonic and volcanic processes. We have performed b-value analysis for the March 2014 earthquake swarm and resulting in an average b-value of 1.39 (see Supplementary Fig. S3). Generally, low b-values (< less than unity) suggest tectonic domain^55^ and larger values (> 1 to 3) suggest volcanic activity^2^. The high b-value observed here may be indicative of magmatic origin of these events.

We observed for the first time that the hydro-acoustic waves are also closely associated with high frequency onset earthquakes in the Andaman–Nicobar region. The occurrence of the hydro-acoustic phase confirms that the events originate at shallow depths (< 30 km), as obtained from the hypocenters of high-frequency onset events (Fig. 4b). We have analysed the T-wave rise time to infer earthquake depths and also to look for any evolutionary behaviour with time. During the initial part of swarm, we observed a rapid decrease in rise time from 35 to 5 s, indicating the migration of hypocentres from deeper to shallower depths probably associated with upward movement of magma. In addition, the average rise time is of the order of 20 s, indicating a relatively deeper source location (Fig. 4c). In the present study, we do not observe any T-wave with small rise time (< 2 s), suggesting seafloor eruption activity is unlikely. The decrease in rise time has been attributed to movement of magma dike intrusions through rift zones of Juan de Fuca and mid-Atlantic ridge spreading centers^18,19^. The hydro-acoustic phase locations are also distributed linearly in the northwest-southeast direction along the inner volcanic arc (Fig. 5d). T-waves (equivalent to hydro-acoustic phase in ocean) can originate due to shallow crust double-couple source mechanism^45,56–58^. Dziak (2001)^57^ proposed that the chances of releasing the hydro-acoustic wave energy into the water column are more for strike-slip fault earthquakes as compared to normal and reverse fault events.

Swarms of earthquakes with T-phase are considered to be a good indicators of magma movement beneath the crust^8,9^. The high-resolution bathymetry data showed several volcanic edifices/topographic highs in the off Nicobar region (Fig. 5a). Further, cratered submarine volcanoes also suggest recent volcanism in this region^23,59^. Previous morphological and geochemical studies of the seabed samples of cratered seamount in this region suggest that the seamount has erupted in the recent (past few hundred years) geological past^23^. Rocks from the off Nicobar volcanic arc have shown geochemical characteristics conforming to calc-alkaline melts derived from lower oceanic crust^60^. The occurrence of the hydro-acoustic phase associated with hybrid VLPEs from off Nicobar submarine volcanoes suggests subsurface magma migration from deeper to shallow depths. In a recent study on the earthquake clusters in the Andaman Sea, Špičák and Vaněk, (2013)^61^ suggested that off Nicobar earthquake swarms are induced by episodes of magma ascent from deeper magma reservoir to the shallow magma chamber. We suggest that March 2014 swarm was generated by the reactivation of shallow fault system connected to a magma reservoir at depth. The upward migration of fluid from the deep magma reservoir due to tectonic forces might have led to earthquakes constituting the swarm. The sliver strike-slip fault system might have provided a natural pathway for upwelling fluids and magmas.

## Conclusions

The off Nicobar region has been witnessing frequent swarms after the 2004 megathrust earthquake. One such earthquake swarm was recorded on 21st and 22nd March 2014 by a passive OBS experiment in the Andaman–Nicobar region. Based on the analysis of this data, we report for the first time hybrid VLPEs which are considered to be an indicator of subsurface magmatic activity. The following conclusions are drawn.

1. The onset of waveform shows a high-frequency (1–10 Hz) earthquake event which is followed by a long-period waveform up to 600 s. The occurrence of VLPEs indicates the subsurface movement of magma near the inner volcanic arc.
2. The onset earthquakes occur at shallow depth and the focal mechanisms indicate right-lateral strike-slip faulting. The epicentres are distributed along the Seulimeum Fault.
3. We also observed hydro-acoustic phase (10–40 Hz) associated with high frequency onset earthquakes. The earthquake swarms and associated hydro-acoustic phase indicate subsurface tectonic and magmatic influence in the region. We suggest strike-slip movement of sliver fault facilitated by the upward movement of magma pulses from deeper magma reservoir to the shallow magma chamber induced earthquake swarms along pre-existing faults in the off Nicobar seismogenic zone.

## Supplementary information

Supplementary Information.
