# Supplementary material for: Sub-surface magma movement inferred from low-frequency seismic events in the off-Nicobar region, Andaman Sea
Source: Sci Rep. 2020 Dec 4;10:21219. doi: 10.1038/s41598-020-78216-2 (PMC7719189; doi:10.1038/s41598-020-78216-2)
Supplement: Supplementary file 1 — Supplementary Information. [file 41598_2020_78216_MOESM1_ESM.pdf]

Scientific Reports

Supplementary Information for

Sub-Surface Magma Movement Inferred from Low-Frequency Seismic Events in the off-Nicobar Region, Andaman Sea

K.K. Aswini[1,2], Pawan Dewangan[1], K.A. Kamesh Raju[1,3,*], V. Yatheesh[1], Pabitra Singha[1], Lalit Arya[1], T. Ramakrushana Reddy[1]

[1]*CSIR-National Institute of Oceanography, Dona Paula, Goa 403004, India*

[2]*School of Earth, Ocean and Atmospheric Sciences, Goa University, Taleigao Plateau, Goa 403206, India*

[3]*ESSO-National Centre for Polar and Ocean Research, Ministry of Earth Sciences, India, Vasco-da-Gama, Goa 403804, India*

*Corresponding author. E-mail: kameshraju@gmail.com, kamesh@ncpor.res.in

Contents of this file

Figures S1 to S3

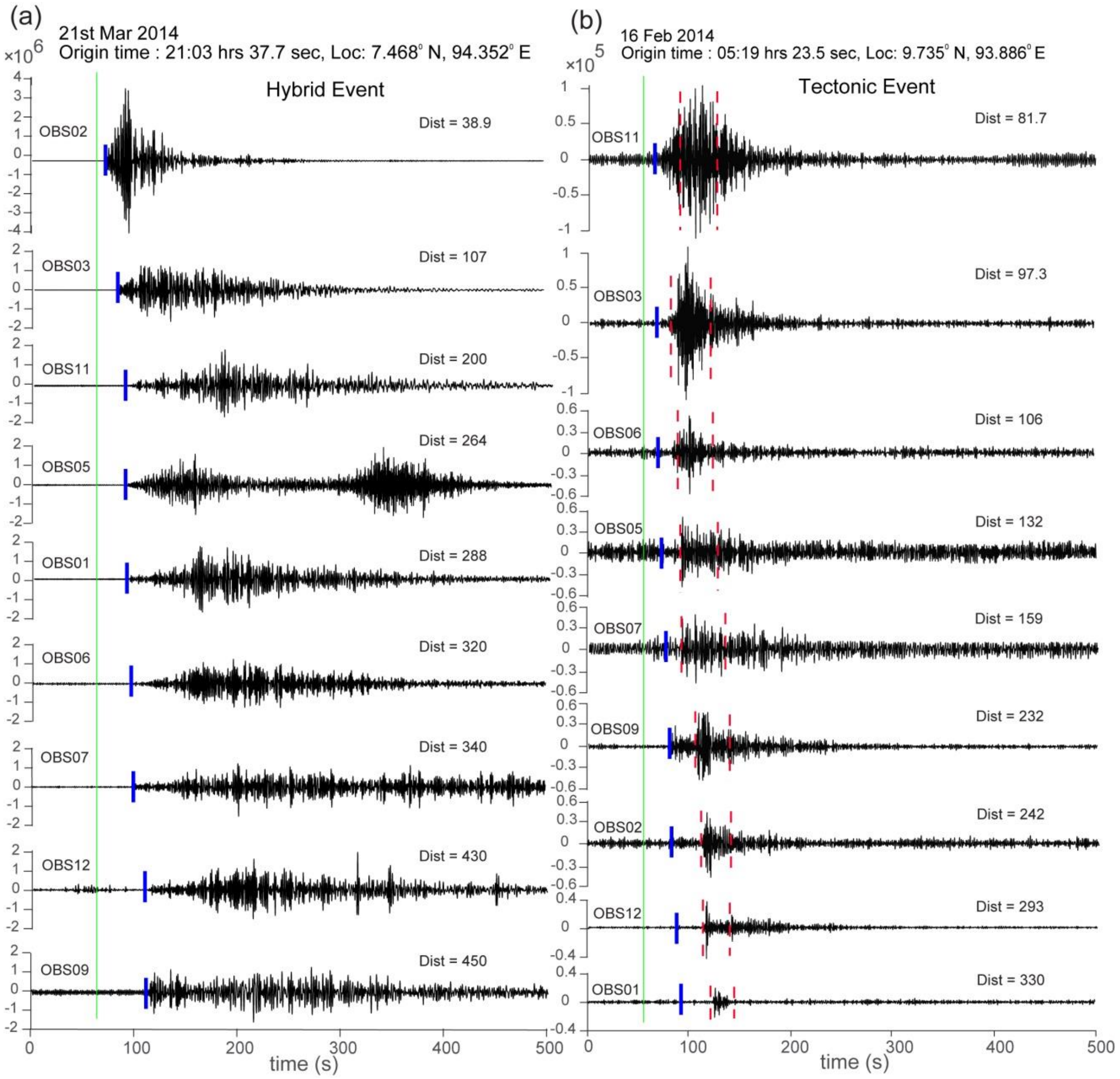

Figure S1. Comparison of waveform characteristics of earthquake event of M_L 4.1 magnitude from off Nicobar swarm region and Andaman Nicobar Fault region as recorded in OBS receivers (locations in Figure 1). The velocity records are normalized by the peak of the trace and a 0.01-1 Hz filter was applied. (a) Waveform of M_L 4.1 hybrid earthquake event of 21st March 2014 in the Swarm region (Location: 7.468 ° N, 94.352 ° E). Prolonged low frequency component present in the hybrid event can be seen. Green line indicates the origin time and the blue lines mark the p-phase. (b) Waveform of M_L 4.1 tectonic earthquake event of 16th February 2014 (Location: 9.735 ° N, 93.886 ° E). Red dashed lines indicate the surface wave window. Note the amplitude of the hybrid event is ten times larger than the tectonic event (Y-axis, normalized amplitude x10^6 for hybrid event).

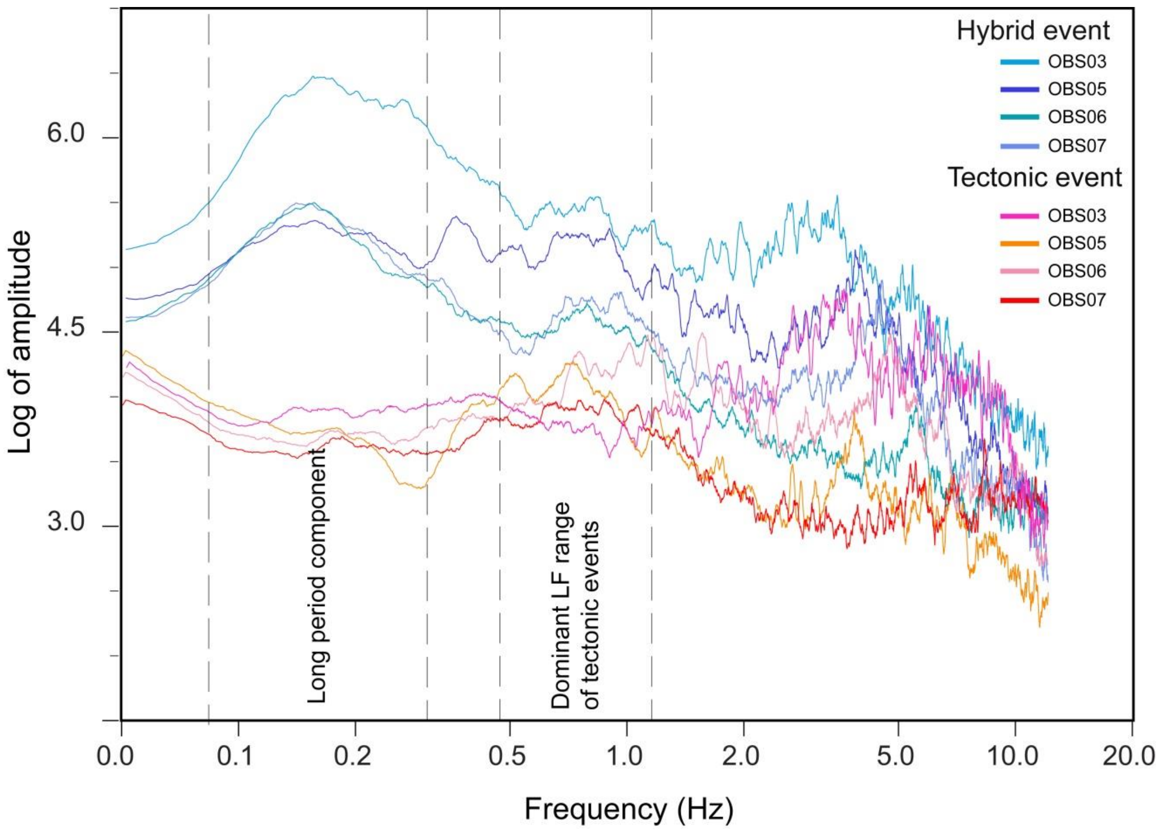

Figure S2. (a) Example of velocity spectra performed for events 21st March 2014, 21:03 hrs. 37.7 sec (M_L 4.1) hybrid earthquake and 16th February 2014, 05:19 hrs 23.5 sec (M_L 4.1) pure tectonic earthquake. Long period window and dominant low frequency range of tectonic events are marked. The dominant frequency range of hybrid events is approximately 0.01-0.3 Hz. These spectra are for entire waveform.

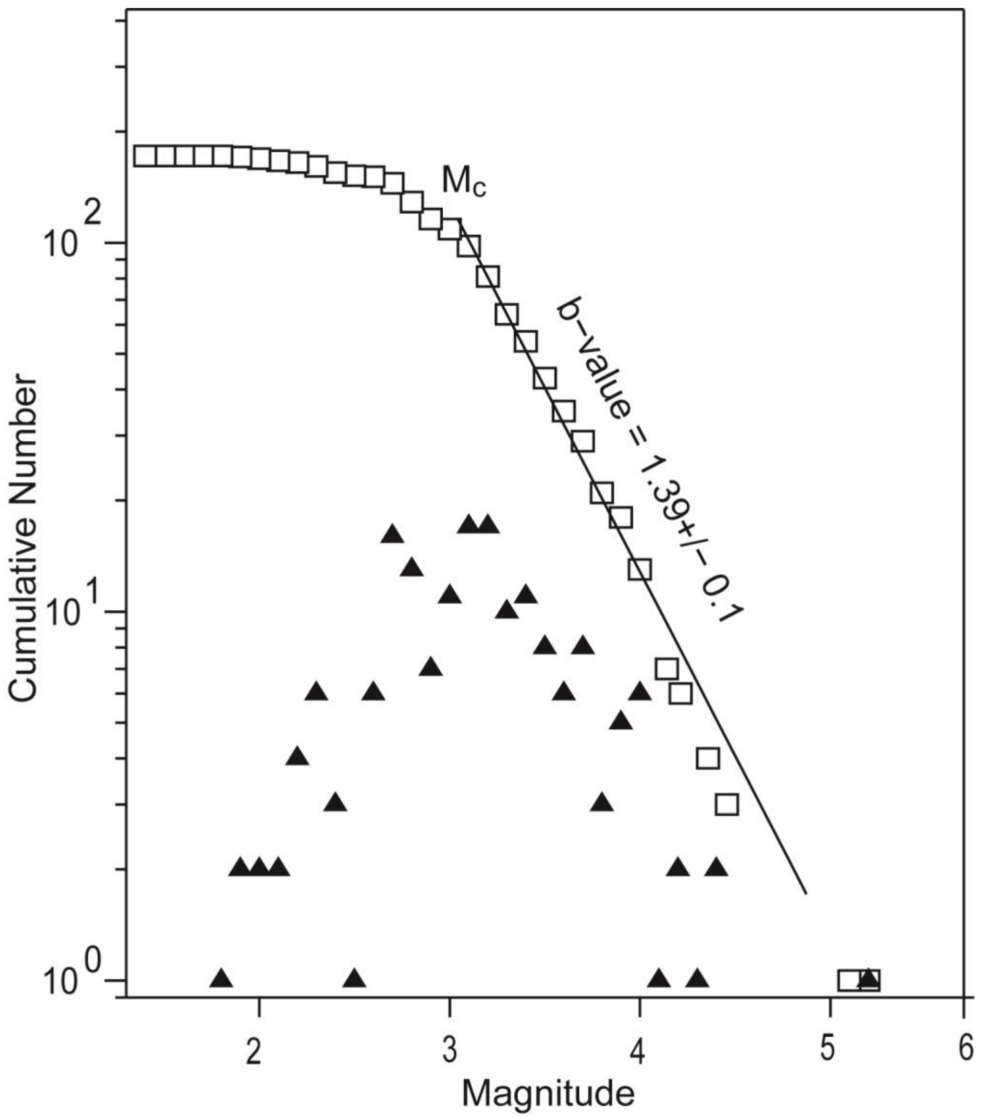

Figure S3. Cumulative magnitude-frequency and b-value of March 2014 swarm. We estimated the b-value using maximum likelihood method[1,2] using Zmap software[2]. For calculating b-value distribution for the earthquake swarm we have chosen the maximum curvature fit of magnitude of completeness value (M_c). Squares and triangles represent cumulative counts and per-bin counts, respectively.

Reference

1 Aki, K. Maximum likelihood estimate of b in the formula log N = a-bM and its confidence limits. *Bull. Seismol. Soc. Am.* **43**, 237-239 (1965).

2 Wiemer, S. A software package to analyze seismicity: ZMAP. *Seismol. Res. Lett.* **72**, 373-382 (2001).
